# Reverse Transrectal Stapling Technique Using the EEA Stapler: An Alternative Approach in Difficult Reversal of Hartmann’s Procedure

**DOI:** 10.4103/2006-8808.73618

**Published:** 2010

**Authors:** Sanoop K. Zachariah

**Affiliations:** *Department of Surgery, MOSC Medical College, Kolenchery, Cochin, India*

**Keywords:** Colorectal anastomosis, diverticulitis, Hartmann’s procedure, EEA stapler, reverse stapling technique

## Abstract

The introduction of circular end-to-end stapling devices (CEEA OR EEA stapler) into colorectal surgery have revolutionised anastomotic techniques. The EEA stapler is generally regarded as an instrument that is safe, reliable, and simple to operate. Despite it’s popularity, very little information is available regarding the technical difficulties encountered during surgery. The routine technique to perform an end-to-end circular colonic anastomosis is to introduce the instrument distally through the anus (transrectal/transanal approach) and attach it to the anvil which is purse stringed at the distal end of the proximal bowel to be anastomosed. Two cases of reversal of Hartmann’s procedure for perforated diverticulitis are described in the present study, where difficulty was experienced while using the EEA stapler in the routine method. Hence, an alternative reverse technique which was used is presented.

## INTRODUCTION

The use of EEA staplers has greatly facilitated the restorative procedures in colorectal surgery, including reversal of Hartmann’s.[1] Despite the recent popularity with the use of mechanical staplers in colorectal surgery, very little information is available regarding the technical difficulties encountered during surgery. Reported problems usually highlight immediate and late complications associated with stapled anastomosis.[1,2] Adam[3] described a technical difficulty where in the EEA stapler got entrapped following the anastomosis and also described a novel technique to retrieve it. In the present study, two cases of reversal of Hartmann’s procedure for perforated diverticulitis, are described, where in difficulty was experienced while using the EEA stapler in the usual manner. Hence, an alternative reverse technique which was used is presented.

## CASE REPORTS

### Case 1

**Figure 1:**
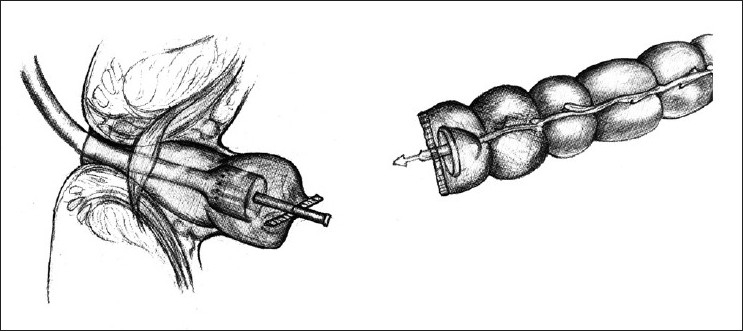
The anvil is secured in the proximal colon while the instrument is introduced transanally—the standard approach

**Figure 2:**
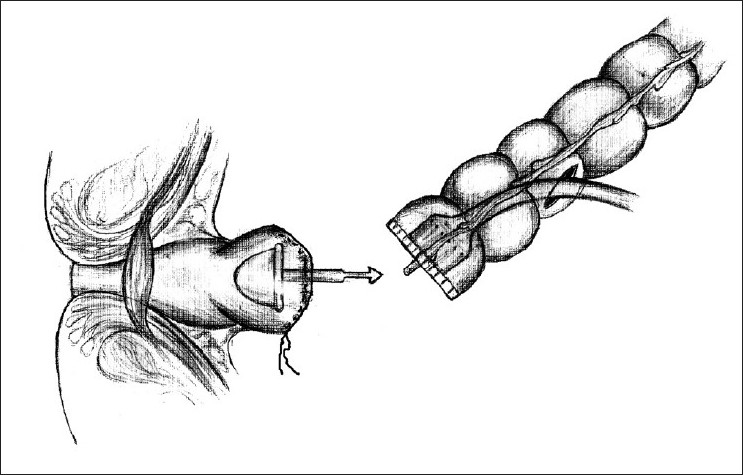
Reverse application of the anvil into the Hartmann’s pouch and the instrument inserted through a vertical colotomy into the proximal colon

**Figure 3:**
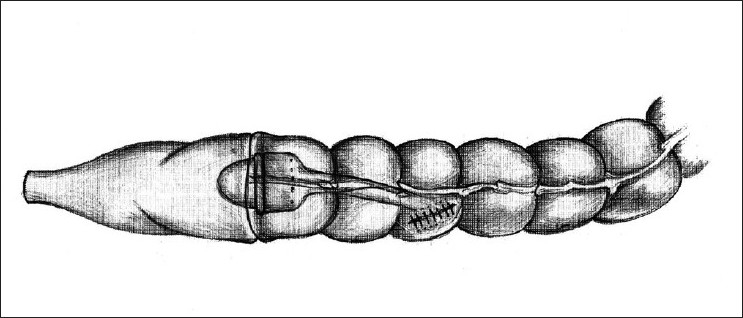
End to end colorectal anastomosis after firing the EEA stapler

A reversal of Hartmann’s procedure was planned for a case of perforated sigmoid diverticulitis, two months after the initial procedure. The patient was a 47-year-old man who presented with peritonitis and shock, for which an emergency exploratory laparotomy was done. During this second stage procedure, it was initially planned on for a reversal Hartmann’s, with restoration of intestinal continuity using an EEA stapler. The routine initial operative steps included release of the colostomy and mobilization of the proximal and distal bowel segments. The distal colon required scrupulous dissection in order to be isolated, because of dense perirectal adhesions. The distal rectal stump is usually identified with the help of a long polypropylene suture, which is tagged to it during the initial surgery. The usual technique involves securing the anvil at distal end of the proximal colon within a purse string suture [Figure 1]. A sizer is used to predetermine the size of the EEA stapler to be used. The instrument is introduced transanally until it reaches the tip of the rectal stump, previously closed with a linearly stapled line, and a rectotomy is made above or below the staple line to allow easy advancement of the central shaft. The anvil’s shaft is then attached to the central shaft and EEA stapler is closed, activated, and fired. In this case, despite of multiple attempts, the EEA stapler could not be advanced smoothly and sufficiently enough to reach the proximal end of the rectal stump. It got stuck somewhere midway between the anus and the tip of the Hartmann’s pouch. After several futile attempts and gentle manipulations of the instrument, it was decided not to try further and negotiate the instrument transanally, due to risk of traumatising the anorectal mucosa. A sigmoidoscopy was done which did not reveal any intraluminal mechanical cause for obstruction. Hence, it was decided to try the reverse technique. The anvil was removed from the proximal segment and purse stringed into the proximal end of the Hartmann’s pouch after excising the stapled line. This could be performed more easily than expected. Subsequently, the distal end of the proximal bowel was closed linearly with hand sewn sutures. A vertical colotomy of 3 cm was made over the tinea coli of the proximal colon, about 10 cm above its distal end [Figure 2]. The EEA instrument was introduced through it, and in this way the two ends could be easily approximated and the stapler fired to make a circular end-to-end inverting anastomosis [Figure 3]. The doughnuts were complete and integrity was checked by under-water air insufflation technique via a Foleys attached to a Toome syringe. The postoperative period was uneventful and the patient is on regular follow-up since past 5 years and is doing well.

### Case 2

Three years later, a 67-year-old man presented with clinical and radiological features of perforation peritonitis. On exploratory laparotomy, a low sigmoid diverticular perforation was found and a Hartman’s performed. Two months later, a reversal was planned. During this surgery, the same difficulty of passing the stapler transrectally was encountered, as in the above case. As in the previous case, there were considerable perirectal adhesions which required meticulous dissection. After multiple futile attempts, a reverse stapling technique was attempted based on the previous experience. The similar steps were repeated and the anastomotic integrity confirmed. Postoperatively, the patient is doing well and is on regular follow-up since two years.

## DISCUSSION

The concept of mechanical stapling devices in clinical surgery was first introduced by a Hungarian surgeon Humer Hult in 1908.[4] The early instruments were complex and cumbersome to use, but were refined over time. With this instrument, an anastomosis can be achieved at a lower level than would be feasible with conventional hand sewn techniques. In addition, it is much more easily and rapidly performed, especially in patients with a narrow and deep pelvis. The original technique for low rectal end-to-end anastomosis with EEA stapler was described by Ravitch and Steichen in 1979.[5] The standard technique for reversal of Hartmann’s routinely practiced and preached procedure is similar to the technique described by Mittal and Cortez.[6]

As with Hartmann’s reversal, restoration of intestinal continuity in patients with long rectal pouches usually does not present technical problems. However, it can be very difficult in patients with short pouches that are retracted or buried deep in the pelvis.[7] It is interesting that the same mechanical difficulty was faced in both these cases of Hartmann’s reversal for perforated diverticulitis. The exact reason is uncertain. Hypoplasia is known to occur in the defunctioned rectum.[8] Other reasons could be some anatomical abnormality of the pelvis unfavorable to the contour of the EEA or a primarily narrow rectum that would have been responsible for diverticulosis in the first place. The difficulty could also be due to dense perirectal inflammatory cicatrix in the pelvis. Such technical difficulties are probably under-reported. However, the sigmoidoscope could be easily passed up to end of the distal stump.

When faced with such a tricky situation on table, there would probably be only two options to pursue. One would be to abandon the idea of using a mechanical stapler and proceed with a hand sewn anastomosis. The second would be to discard the stapler in use and employ a smaller-sized stapler. In both cases, the choice would entail an additional financial burden to the patient.

The technique described here is similar to the transabdominal approach, but was not popular as it necessitated a second colotomy closure.[9,10] The advantage here is that the same instrument can be used, and the difficulties associated with hand sewn technique can be avoided. The disadvantage involves need for a colotomy and an inherent risk for its disruption. However in both these cases, there was no clinical evidence of loss of bowel integrity. In both the cases, the vertical colotomy was closed in two layers and reinforced with an omental patch.

It is therefore felt that the technique described here would benefit surgeons who might be faced with such a difficult situation. Since the method involves a reverse application of the anvil, namely into the distal loop and the gun through the proximal loop via a colotomy, the term reverse transrectal stapling is used.

## CONCLUSION

Reverse transrectal stapling can be a useful alternative technique of performing reversal of Hartmann’s with an EEA stapler when the instrument cannot be passed through the anus. It can save time of otherwise performing a hand sewn anastomosis and save cost of using a second instrument. Further work on technical difficulties need to be addressed and reported by surgeons and researched by manufacturers.

## References

[1] Marti MC, Fiala JM, Rohner A (1981). EEA stapler in large bowel surgery. World J Surg.

[2] Nance FC (1979). New techniques of gastrointestinal anastomoses with the EEA stapler. Ann Surg.

[3] Adams DB (1989). The entrapped EEA TM stapler: backing out with ease. Dis Colon Rectum.

[4] Robicsek F, Konstantinov I (2001). Humer Hultl: The father of the surgical stapler. J Med Biogr.

[5] Ravitch MM, Steichen FM (1979). A stapling instrument for end-to-end inverting anastomosis in the gastrointestinal tract. Ann Surg.

[6] Mittal VK, Cortez JA (1981). Hartmann procedure reconstruction with EEA stapler. Dis Colon Rectum.

[7] Caracciolo F, Castrucci G, Castiglioni GC (1986). Anastomosis with EEA™ stapler following Hartmann procedure. Dis Colon Rectum.

[8] Appleton GV, Williamson RC (1989). Hypoplasia of defunctioned rectum. Br J Surg.

[9] Beart RW, Wolff BG (1982). The use of staplers for anterior anastomoses. World J Surg.

[10] Ragins H, DeLuca FR (1981). Technique for using the EEA stapler for low anterior resection of the rectum entirely from the abdominal approach. Am J Surg.

